# Akzidentielle Injektion eines unbekannten Notfallantidots zur Acetylcholinesteraseaktivierung

**DOI:** 10.1007/s00113-021-01010-w

**Published:** 2021-05-25

**Authors:** Sebastian Zinn, Ingo Marzi, Maren Janko

**Affiliations:** 1 Klinik für Anästhesiologie, Intensivmedizin und Schmerzmedizin, Universitätsklinikum, Goethe-Universität Frankfurt, Theodor-Stern-Kai 7, 60590 Frankfurt, Deutschland; 2 Klinik für Unfall‑, Hand- und Wiederherstellungschirurgie, Universitätsklinikum, Goethe-Universität Frankfurt, Theodor-Stern-Kai 7, 60590 Frankfurt, Deutschland

## Falldarstellung

### Anamnese

Eine 61 Jahre alte Patientin wird notarztbegleitet nach akzidentieller Injektion eines dem Behandlungsteam unbekannten Antidots übernommen. Es wird übergeben, dass sich die Patientin während der Arbeitszeit eine unbekannte Menge des Stoffes Pralidoximchlorid injiziert habe. Bei dem Stoff handelt es sich um ein militärisch genutztes Antidot, dass u. a. für den Fall des Einsatzes von chemischen Waffen (z. B. Sarin, VX, Nowitschokverbindungen) vorgehalten wird, die durch Hemmung der Acetylcholinesterase zur Beeinträchtigung synaptischer Übertragungen führen. Die Patientin arbeite in einer U.S.-Einrichtung, in der Autoinjektoren für Notfallsituationen mit diesem Stoff vorgehalten werden. Bei der routinemäßigen Entsorgung der Injektoren kam es zur Auslösung des Injektionsmechanismus. Die Injektion des Pralidoximchlorids erfolgte dabei versehentlich in den Daumen der linken Hand. Die Patientin klagte anfänglich über Flush-Symptomatik und stärkste Schmerzen (NRS 10/10) an der Injektionsstelle. Der alarmierte Rettungsdienst überstellte die Patientin in die nächstgelegene Klinik zur Akutversorgung. Dort erfolgten frustrane Analgesieversuche mit 1 g Paracetamol und 7,5 mg Piritramid. Bei Aggravation der Schmerzen erfolgte eine orale Therapie mit Oxycodon, 5 mg, und zuletzt mit 20/10 mg Oxycodon/Naloxon, ebenfalls ohne suffizientes Analgesieergebnis. Der kontaktierte Giftnotrufdienst teilte mit, dass der Wirkstoff im deutschsprachigen Raum nicht zugelassen sei und keine Erfahrungswerte existierten. Es wurde eine lokale Wärmetherapie empfohlen; eine vasodilatative Therapie wurde nicht angeraten [5]. Bei zunehmend livider Verfärbung des Daumens und anhaltenden Schmerzen entschieden sich die Kollegen zur notarztbegleiteten Verlegung der Patientin in ein Haus der Maximalversorgung.

### Klinischer Aufnahmebefund

Die Patientin ist wach, kreislaufstabil, normofrequenter Sinusrhythmus und zu allen Qualitäten adäquat orientiert. Sie gibt weiterhin stärkste, undulierende Schmerzen (NAS 9/10) im Bereich des linken Daumens an. Motorik ist, soweit beurteilbar, intakt. Es wird von einem zunehmenden Taubheitsgefühl im Bereich des Daumens berichtet. Die Patientin klagt zudem über ein unangenehm trockenes Gefühl im Mund.

**Figure 1:**
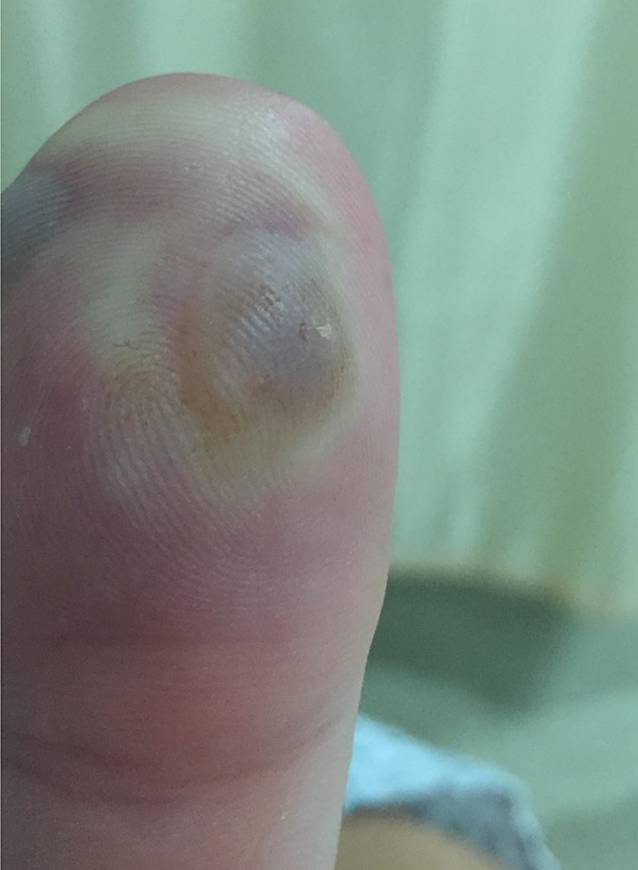

An der Injektionsstelle zeigen sich 2 flüssigkeitsgefüllte, pralle Blasen mit perifokal leichter Abblassung. Die Haut ist sonst intakt, keine Wunden, keine Nekrosen (Abb. 1).

Im Gespräch mit der Patientin berichtet diese, dass es sich bei dem Injektor um einen Notfallinjektor aus dem militärischen Bereich handelt, der für den Fall des Einsatzes von chemischen Waffen/Kampfgasen in der Einrichtung vorgehalten werde.

### Diagnose

Verätzung des Daumens von weniger als 10 % der Körperoberfläche.

## Therapie und Verlauf

### Prozedere in der Notaufnahme

Es erfolgt zunächst die Kontaktaufnahme mit dem Bundeswehrkrankenhaus in Koblenz. Die dortigen Kollegen stellen einen Kontakt zum Army Medical Center in Landshut her. Dort erfolgte die Beratung durch Toxikologen der US Army. Es wurden eine 4‑stündige Monitorüberwachung, lokale Wärmetherapie, ggf. RR-Einstellung mit Nitrospray/Urapidil oder anderen geeigneten Medikamenten empfohlen. Außerdem wurde bei anhaltender Verfärbung des Fingers nach lokaler Wärmetherapie oder bei Nichttolerierbarkeit durch die Patientin eine lokale vasodilatative Therapie mit Nitrospray empfohlen.

Da ein Wärmekontakt am Finger nicht toleriert wird, erfolgt die Gabe von 1 Hub Nitrospray auf die Daumenkuppe unter fortlaufender Monitorkontrolle. Es stellt sich innerhalb weniger Minuten eine deutliche Schmerzlinderung von NAS 9 auf NAS 3 ein.

Im Aufnahmelabor zeigt sich ein unauffälliger Befund. Die venöse Blutgasanalyse ergibt einen ausgeglichener Säure-Base-Haushalt mit einem milden Anstieg des Lactats auf 24 mg/dl (Ref.: 4,5–14,5 mg/dl).

Die Patientin wird hämodynamisch weiter überwacht. Im Verlauf zeigen sich ein Blutdruckanstieg bis sys. 180 mm Hg, der durch Hausmedikation suffizient gesenkt werden kann. Es folgt nach 4 h eine stationäre Überwachung und am Folgetag die Entlassung in die ambulante Behandlung.

### Befund im Verlauf

**Figure 2:**
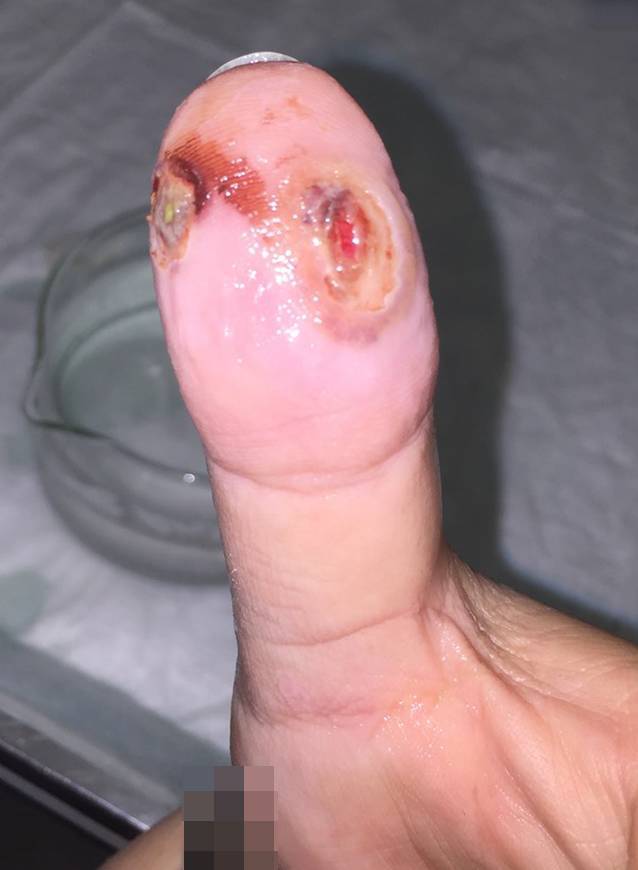

Im Verlauf zeigte sich die Blase wenig rückläufig, sodass eine chirurgische Wundabtragung erfolgte (Abb. 2). Die Wunde verheilte nach 6 Wochen. Es blieb eine leichte Dysästhesie im Verlauf bis 14 Wochen bestehen, die jedoch rückläufig erscheint, ohne Beeinträchtigung.

## Diskussion

Pralidoximchlorid gehört zur Substanzklasse der Acetylcholinesteraseaktivatoren. Die Indikation für die Substanzklasse besteht als Antidot für Organophosphatvergiftungen [2]. Organophosphate, wie das Pestizid E306, aber auch chemische Nervenkampfstoffe wie Tabun, Sarin oder Nowitschokverbindungen führen zu einer Hemmung der Acetylcholinesterase über Anheftung einer organischen Phosphatgruppe, die zum „aging“ des Enzyms und zu einer funktionslosen Acetylcholinesterase führt. Dies betrifft sowohl die neuronalen Acetylcholinesterasen als auch die Butyrylcholinesterasen im Blut. Infolgedessen kommt es zum Anstieg der Acetylcholinkonzentration im Zentralnervensystem und im peripheren Nervensystem. Sowohl das motorische als auch das autonome Nervensystem sind betroffen. Es kommt akut zu einem Depolarisationsblock der Muskulatur, inkl. Atemmuskulatur, und vegetativer Dysregulation. Zentrale Symptome zeigen sich in Form von Bewusstseinsverlust, Minderung des Atemantriebs. Autonome Symptome können sowohl als muskarinerge Effekte mit Bradykardie, Bronchospasmen, Übelkeit und Erbrechen sowie als nikotinerge Effekte mit Hypertension, Tachykardie und Schwitzen imponieren [3].

Antidote der Oximgruppe wie Pralidoxim katalysieren die Spaltung dieser Organophosphatbindung und stellen die Funktion der Acetylcholinesterase wieder her. Im Vergiftungsfall werden als Erstmaßnahme die Gabe von Atropin, 2 mg i.v./i.m. (max. 50 mg), empfohlen. Oxime sollten so zeitnah wie möglich appliziert werden. In Deutschland steht über Notfalldepots Obidoxim zur Verfügung; Pralidoxim steht nicht flächendeckend zur Verfügung [1, 9].

Die alleinige, systemische Anwendung von Oximen, ohne Organophosphatvergiftung, führte in kleinen Studien zu sympathikomimetischen Effekten, wie sie auch bei der Patientin beobachtet wurden. Es kam zu Anstiegen des Blutdruckes und der Herzfrequenz sowie antimuskarinergen Symptomen wie Mundtrockenheit und Hitzegefühl. Ein sympathikomimetischer Effekt durch lokale, hohe Konzentrationen an der Einstichstelle bietet auch die Erklärung für die Minderperfusion im betroffen Endstromgebiet als Folge einer lokalen Vasokonstriktion. Diese Annahme stellte auch die Rationale für die Entscheidung zur lokalen, vasodilatativen Therapie da. Dieser Mechanismus wird durch das gute therapeutische Ansprechen untermauert. Die starken Schmerzen interpretierten wir daher vorranging als Ischämieschmerz. Über den genauen Mechanismus der Vasokonstriktion kann nur gemutmaßt werden. Es liegt die Hypothese nahe, dass eine Wirkung des Oxims – auch ohne Reaktivierung einer Organophosphatbindungsstelle – zu einem „überphysiologischen“ Abbau von Acetylcholin führt und in der Folge eine sympathikomimetische Wirkung mit Vasokonstriktion überwiegt [6, 9].

Der dargestellte Fall ist in dieser Form sicherlich eine Rarität. Er zeigt jedoch auf, dass in zeitlich kritischen Situationen ungewöhnliche Informationswege genutzt werden können, um eine Therapie zu ermöglichen. Ohne die Rückmeldung durch das Militärhospital wäre eine Therapie weiter verzögert worden. Ob eine noch zeitigere Informationsbeschaffung zwischen Unfall und letztlich erfolgreicher Therapie den individuellen Heilungsverlauf begünstigt hätte, bleibt Spekulation.

Des Weiteren zeigt der Fall auf, dass die Konfrontation mit ungewöhnlichen Intoxikationen im Rahmen der Katastrophenmedizin jederzeit in den ungewöhnlichsten Formen präsent werden kann. Auch wenn in der hier geschilderten Kasuistik ein Unfall zur Konfrontation mit der Thematik der Organophoshate bzw. deren Antidote führte, ist eine Konfrontation von Unbeteiligten, wie im Fall von Salisbury 2018 zwar sehr unwahrscheinlich, aber nicht ausgeschlossen [4, 8]. Im Zusammenhang mit dem Fall des russischen Politikers Alexei Nawalny wird ebenfalls eine Behandlung mit Pralidoxim und Atropin genannt [7].

## Fazit für die Praxis

Der beschriebene Fall imponiert v. a. durch die Konfrontation mit dem Thema der Vergiftungen durch militärisch genutzte Stoffe und die Tatsache, dass in der Akutsituation dem zivilen Arzt nur unzureichende Informationsquellen zur Verfügung stehen.

Der Bericht soll daher v. a. mögliche, ungewöhnliche Wege der Informationsbeschaffung aufzeigen. Die Kommunikation mit den militärischen Einrichtungen verlief freundlich und zielführend.

Insbesondere da in den letzten Jahren in Europa, und nun auch in Deutschland, Substanzen aus den Bereichen der Organophosphate in Einzelfällen Aufsehen erregt haben, ist die zeitnahe Beschaffung von Informationen von tagesaktueller Bedeutung.

## Abkürzungen

2‑PAM Cl: Pralidoximchlorid (Protopam Chlorid®)
